# Recurrent symmetrical drug-related intertriginous and flexural exanthema caused by different iodinated contrast media

**DOI:** 10.1186/2045-7022-5-S1-O1

**Published:** 2015-03-11

**Authors:** María Elisa Caralli, Dasha Roa Medellin, Manuel De Barrio Fernández

**Affiliations:** 1 Hospital General Universitario Gregorio Marañón, Allergy Department, Madrid, Spain

## Background

Symmetrical drug-related intertriginous and flexural exanthema (SDRIFE), is a self-limiting type IV hypersensitivity reaction characterized by sharply defined symmetric erythema involving the gluteal area and the flexural or intertriginous folds without any systemic symptoms. SDRIFE caused by iodinated contrast media (ICM) have been rarely reported.

## Method

A 76-year-old woman with the diagnostic suspicion of urinary lithiasis, was subjected to a CT scan with an unknown ICM.

Forty-eight hours after the CT scan was carried out, she developed an erythema in both axillae, inframammary folds, cubital and popliteal fossae, such as gluteal and inguinal area, without systemic symptoms. She was treated with methylprednisolone and she began to improve one week later.

The patient had no previous history of drug hypersensitivity and she had tolerated ICM six years before.

She was referred to our Drug Allergy Unit for study, where epicutaneous and single-blind intravenous controlled challenge tests were performed.

## Results

Intradermal and epicutaneous tests were led with iomeprol, iopamidol and ioversol being negative.

A single blind intravenous controlled challenge test with iomeprol was performed after the patient filled in an informed consent. Forty-eight hours after the administration of 30 ml the patient developed a sharply erythema of the inframammary folds, inguinal area, and cubital and popliteal fossae, without systemic symptoms.

Four and eight weeks later respectively, single-blind intravenous controlled challenge tests with iopamidol (30 ml) and ioversol (30 ml) were performed, and the patient had similar symptoms.

## Conclusions

We present a case of SDRIFE due to iomeprol, iopamidol and ioversol confirmed by positive controlled challenge tests.

In our case, intradermal and epicutaneous test were not useful, therefore, controlled challenge test are necessary as have been demonstrated in other cases.

Cross-reactivity was demonstrated between three different ICM in controlled challenge tests.

## Consent

Written informed consent was obtained from the patient for publication of this abstract and any accompanying images. A copy of the written consent is available for review by the Editor of this journal.

